# Marital status as an independent prognostic factor for patients of malignant pleural mesothelioma

**DOI:** 10.3389/fmed.2022.955619

**Published:** 2022-10-21

**Authors:** Shu Pan, Na Yan, Yuanyuan Zhao, Zhiwen Li

**Affiliations:** Department of Anesthesiology, The First Hospital of Jilin University, Changchun, Jilin, China

**Keywords:** malignant pleural mesothelioma, marital status, SEER, survival, cancer specific survival, overall survival

## Abstract

**Objectives:**

The prognostic impact of marital status on malignant pleural mesothelioma (MPM) is not investigated. This paper probes into the relationship between the prognosis of MPM and marital status.

**Materials and methods:**

The Surveillance, Epidemiology, and End Results (SEER) database of American had been applied to choose eligible patients over the 2004–2015 periods. Moreover, cancer-specific survival (CSS) and overall survival (OS) of unmarried and married groups were compared.

**Results:**

A total of 3,997 patients in total had been identified, including 2,735 (68.43%) married patients. In comparison to unmarried patients, married ones tended to be younger, male, white, and received active treatment (surgery, chemotherapy, or radiotherapy). In addition, the 1, 3, and 5-year CSS rates were 44.40, 12.09, and 6.88% in married patients, while 35.75, 12.12, and 6.37% in unmarried group (*p* = 0.0014). At the same time, the 1, 3, and 5-year OS rates were 41.84, 10.56, and 5.91% in married patients, while 33.67, 10.44, and 4.93%, respectively, in the unmarried group (*p* < 0.0001). As revealed by the multivariate analysis results, the marital status was an independent favorable prognostic factor, in which the married groups showed better CSS [hazard ratio (*HR*): 0.870; 95% confidence interval (*CI*): 0.808–0.938; *p* < 0.001] as well as OS (*HR*: 0.871; 95% *CI*: 0.810–0.936; *p* < 0.001). According to the results of subgroup analysis, the CSS and *OS* survival of married groups were better than the unmarried groups in almost all the subgroups.

**Conclusion:**

Marital status is an independent favorable prognostic indicator of MPM. Poor prognosis in unmarried patients is likely to be related to insufficient treatments and socioeconomic and psychosocial factors.

## Introduction

As an aggressive cancer, malignant pleural mesothelioma (MPM) is related to the previous exposure of asbestos, which has a long latency (1–5). Over the past few years, the MPM’s worldwide incidence has increased in a steady way, causing a global burden (1, 2). However, knowledge of MPM is currently limited, and the clinicopathological characteristics and outcome for this entity are not very clear (6).

Malignant pleural mesothelioma’s prognosis is poor, and its median survival is 8–14 months since diagnosis (1–3, 7). In fact, the prognostic factors of MPM have been reported by a lot of studies, and they primarily pay attention to the clinicopathological features, such as American Joint Committee on Cancer (AJCC) tumor–node–metastasis (TNM) stages, treatment, age, and gender (1, 2, 7). At present, the function of social determinant in the disease development is more stressed (8). As claimed by some researchers, the marital status refers to a prognostic factor in several cancers such as pancreatic cancer, breast cancer, lung cancer, melanoma, and prostate cancer (9–13). However, the effect of marital status on the MPM survival has not been studied previously.

The Surveillance, Epidemiology, and End Results (SEER) program includes 18 different cancer registries’ research data, and covers 30% of American population (14). What’s more, the data of SEER have been extensively applied to probe into the connection between survival outcome and marital status in cancer patients (8, 12, 15). This study will use the SEER database to explore the association of marital status with the MPM survival.

## Materials and methods

### Ethics statement

The SEER Research Data Agreement had been signed to acquire SEER information using the reference number of 19828-Nov 2018. Furthermore, we abided by guidelines and got data with the research approaches. In addition, the Human Research Protection Office considered that the data analysis was focused on non-human subjects and they were available. Hence, the approval from the institutional review board was not required.

### Study population

The tool of SEER*State v8.3.6 was adopted to choose the qualified subjects, including 18 SEER areas over the 1998–2015 periods (2018 submission). The standards for inclusion are as follows: (1) it should be primary MPM patients; and (2) MPM confirmed by pathology and diagnosed in line with the International Classification of Disease for Oncology, Third Edition (ICD-O-3; coded as 9050–9053) (16). The standards for exclusion are as follows: (1) patients had multiple primary tumors; (2) the diagnosis source of patients was from death certificate or autopsy, or they were just diagnosed in a clinical manner; (3) patients without survival and prognosis data; (4) patients had no AJCC stage; (5) patients had information of unknown race and marital status; and (6) patients had no surgery information or died within 1 month after surgery. Then, the remaining subjects were recruited as the initial groups of SEER.

### Covariates and endpoint

The patients’ features had been examined through the factors, such as age, gender, histology, marriage status, race, grade, size of tumor, stage of AJCC, surgery, chemotherapy, and radiotherapy. We classified patients as married or unmarried (such as never married single, divorced, separated, and widowed) (9, 17). The grouping of the age referred to the published studies (<50, 50–69, and ≥70) (18). In terms of the race, patients were classified as black, white, and others (12). In terms of the histology, they were classified into biphasic, epithelioid, fibrous, and sarcomatoid type. As for the staging of cancer, all the qualified cases were reorganized based on the eighth AJCC TNM staging system (19).

The endpoint of this study was cancer-specific survival (CSS) and overall survival (OS). CSS was defined as the period from diagnosis to death attributed to MPM. OS was defined as the period from diagnosis to death from any cause. According to the 2018 Submission Database of SEER, the cut-off date was decided in advance, in which the data of death were included. Hence, the cut-off date was determined on November 31, 2018.

### Statistical analyses

Kaplan–Meier (K–M) approach was used for univariate analysis. Meanwhile, the log-rank test was performed to measure the difference between CSS and OS. Beyond that, the variables with *p*-value lower than 0.1 were assessed in the Multivariate Cox Proportional Hazard Model. In the Cox regression analysis, the subgroup analysis was conducted. The SPSS software had been used to make statistical analysis. Additionally, the survival curves and forest plots were produced using the GraphPad Prism 5. It was considered that a two-sided *p* < 0.05 was statistically significant.

## Results

### Patient characteristics

**FIGURE 1:**
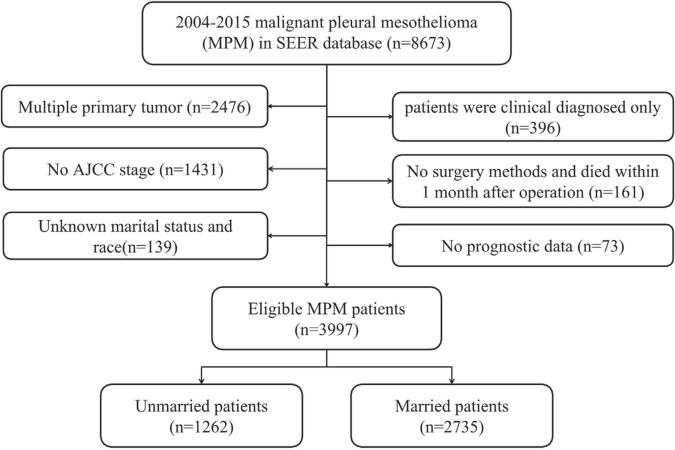
Flowchart showing the patient screening process.

In total, there were 8,673 patients with MPM from 2004 to 2015. In accordance with the standards of exclusion, 3,997 patients have been recruited after screening (see details in Figure 1). Overall, the median survival time was 9.0 months (range: 0–152 months). Then, the included patients were classified into unmarried (*n* = 1,262, 31.57%) and married groups (*n* = 2,735, 68.43%). As for the patients’ baseline features stratified by marital status, they had been showed in Table 1. Additionally, significant difference between unmarried and married groups in the age (*p* = 0.011), gender (*p* < 0.001), race (*p* < 0.001), AJCC stage (*p* = 0.048), surgery (*p* < 0.001), chemotherapy (*p* < 0.001), and radiotherapy (*p* < 0.001) could be observed. Besides, married patients were often younger, male, and white race and they had mostly received surgery, chemotherapy, and radiotherapy. In addition, among the married patients, there was a slightly less stage I/II patients compared to unmarried group (38.83 vs. 40.72%).

**TABLE 1:** The clinicopathological characteristics and treatments of the included 3,997 malignant pleural mesothelioma (MPM) patients.

Characteristic	Total	Unmarried	Married	*P*-value
**Insured status**				0.984
Uninsured/unknown	1027 (25.69%)	324 (25.67%)	703 (25.70%)	
Any medicaid/insured	2970 (74.31%)	938 (74.33%)	2032 (74.30%)	
**Age**				0.011
<50	138 (3.45%)	54 (4.28%)	84 (3.07%)	
50–69	1534 (38.38%)	448 (35.50%)	1086 (39.71%)	
≥70	2325 (58.17%)	760 (60.22%)	1565 (57.22%)	
**Gender**				<0.001
Female	865 (21.64%)	444 (35.18%)	421 (15.39%)	
Male	3132 (78.36%)	818 (64.82%)	2314 (84.61%)	
**Race**				<0.001
Black	209 (5.23%)	95 (7.53%)	114 (4.17%)	
White	3640 (91.07%)	1123 (88.99%)	2517 (92.03%)	
Other	148 (3.70%)	44 (3.49%)	104 (3.80%)	
**Year of diagnosis**				0.982
2004–2017	1277 (31.95%)	402 (31.85%)	875 (31.99%)	
2018–2011	1382 (34.58%)	439 (34.79%)	943 (34.48%)	
2011–2014	1338 (33.48%)	421 (33.36%)	917 (33.53%)	
**Histology**				0.154
Biphasic	345 (8.63%)	99 (7.84%)	246 (8.99%)	
Epithelioid	1597 (39.95%)	501 (39.70%)	1096 (40.07%)	
Fibrous	477 (11.93%)	137 (10.86%)	340 (12.43%)	
Sarcomatoid	1578 (39.48%)	525 (41.60%)	1053 (38.50%)	
**Grade**				0.897
Grade I/II	101 (2.53%)	30 (2.38%)	71 (2.60%)	
Grade III/IV	295 (7.38%)	95 (7.53%)	200 (7.31%)	
Unknown	3601 (90.09%)	1137 (90.10%)	2464 (90.09%)	
**Tumor size**				0.381
<3 cm	287 (7.18%)	91 (7.21%)	196 (7.17%)	
3–7 cm	475 (11.88%)	151 (11.97%)	324 (11.85%)	
≥7 cm	262 (6.55%)	70 (5.55%)	192 (7.02%)	
Unknown	2973 (74.38%)	950 (75.28%)	2023 (73.97%)	
**AJCC stage**				0.048
Stage I	935 (23.39%)	309 (24.48%)	626 (22.89%)	
Stage II	641 (16.04%)	205 (16.24%)	436 (15.94%)	
Stage III	936 (23.42%)	261 (20.68%)	675 (24.68%)	
Stage IV	1485 (37.15%)	487 (38.59%)	998 (36.49%)	
**Type of surgery**				<0.001
Nosurgery	2937 (73.48%)	983 (77.89%)	1954 (71.44%)	
Palliative	752 (18.81%)	204 (16.16%)	548 (20.04%)	
Radical	308 (7.71%)	75 (5.94%)	233 (8.52%)	
**Chemotherapy**				<0.001
No/unknown	1866 (46.69%)	699 (55.39%)	1167 (42.67%)	
Yes	2131 (53.31%)	563 (44.61%)	1568 (57.33%)	
**Radiotherapy**				<0.001
No/unknown	3489 (87.29%)	1135 (89.94%)	2354 (86.07%)	
Yes	508 (12.71%)	127 (10.06%)	381 (13.93%)	

### Marital status and survival

**FIGURE 2:**
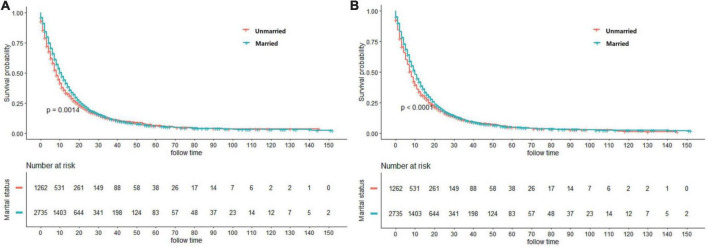
Kaplan–Meier curves showing cancer-specific survival (CSS) **(A)** and overall survival (OS) **(B)** between married and unmarried patients.

In terms of the marital status, CSS and OS survival difference could be observed, as displayed in the Kaplan–Meier curves (Figure 2). To be specific, CSS and OS of married patients were better than those of the unmarried ones. The 1, 3, and 5-year CSS rates were 44.40, 12.09, and 6.88%, respectively, in married groups, while 35.75, 12.12, and 6.37%, respectively, in unmarried groups (*p* = 0.0014). Meanwhile, the 1, 3, and 5-year OS rates were 41.84, 10.56, and 5.91%, respectively, in married groups, while 33.67, 10.44, and 4.93%, respectively, in unmarried groups (*p* < 0.0001). The univariate log-rank test revealed that some covariates had significant association with CSS (*p* < 0.05), which included marital status, histology, gender, age, grade, AJCC stage, size of tumor, surgery methods, chemotherapy, and radiotherapy. Marital status remained a prognostic factor even after multivariate analysis adjustment. Compared with the unmarried groups, married ones had better CSS [hazard ratio (*HR*): 0.870; 95% confidence interval (*CI*): 0.808–0.938; *p* < 0.001]. Meanwhile, all included covariates were significantly associated with OS. According to the multivariate analysis, the marital status remained an independent prognostic factor of *OS* (*HR*: 0.871; 95% *CI*: 0.810–0.936; *p* < 0.001) (Table 2).

**TABLE 2:** Univariate and multivariate analyses of cancer special survival (CSS) and overall survival (OS) for patients with MPM.

Variables	CSS			OS		
		
	Univariate analysis	Multivariate analysis		Univariate analysis	Multivariate analysis	
				
	*P*-value	HR (95% CI)	*P*-value	*P*-value	HR (95% CI)	*P*-value
Insured status	0.644		NI	0.268		NI
Uninsured/unknown						
Any medicaid/insured						
Age	<0.001		<0.001	<0.001		<0.001
<50		Reference			Reference	
50–69		1.276 (1.041, 1.563)	0.019		1.292 (1.061, 1.574)	0.011
≥70		1.610 (1.315, 1.971)	<0.001		1.621 (1.332, 1.973)	<0.001
Gender	<0.001		<0.001	<0.001		<0.001
Female		Reference			Reference	
Male		1.252 (1.149, 1.363)			1.265 (1.164, 1.374)	
Race	0.867		NI	0.904		NI
Black						
White						
Other						
Year of diagnosis	0.413		NI	0.148		NI
2004–2017						
2018–2011						
2011–2014						
Histology	<0.001		<0.001	<0.001		<0.001
Biphasic		Reference			Reference	
Epithelioid		0.646 (0.570, 0.732)	<0.001		0.652 (0.577, 0.737)	<0.001
Fibrous		1.467 (1.263, 1.704)	<0.001		1.474 (1.274, 1.704)	<0.001
Sarcomatoid		0.803 (0.708, 0.912)	0.001		0.816 (0.721, 0.923)	0.001
Grade	<0.001		<0.001	<0.001		<0.001
Grade I/II		Reference			Reference	
Grade III/IV		2.096 (1.609, 2.732)	<0.001		1.927 (1.503, 2.469)	<0.001
Unknown		1.530 (1.129, 1.811)	0.003		1.311 (1.053, 1.632)	0.015
Tumor size	0.060		0.024	0.025		0.015
<3 cm		Reference			Reference	
3–7 cm		0.998 (0.849, 1.173)	0.980		1.019 (0.871, 1.193)	0.811
≥7 cm		1.159 (0.961, 1.398)	0.122		1.197 (0.998, 1.435)	0.052
Unknown		1.143 (1.000, 1.308)	0.051		1.158 (1.016, 1.320)	0.028
AJCC stage	<0.001		<0.001	<0.001		<0.001
Stage I		Reference			Reference	
Stage II		1.301 (1.173, 1.442)	0.185		1.085 (0.976, 1.207)	0.132
Stage III		1.301 (1.173, 1.442)	<0.001		1.295 (1.171, 1.431)	<0.001
Stage IV		1.603 (1.460, 1.760)	<0.001		1.594 (1.456, 1.745)	<0.001
Type of surgery	<0.001		<0.001	<0.001		<0.001
No surgery		Reference			Reference	
Palliative		0.672 (0.612, 0.737)	<0.001		0.682 (0.623, 0.746)	<0.001
Radical		0.560 (0.484, 0.648)	<0.001		0.603 (0.524, 0.692)	<0.001
Chemotheray	<0.001		<0.001	<0.001		<0.001
No/unknown		Reference			Reference	
Yes		0.703 (0.655, 0.754)			0.684 (0.638, 0.732)	
Radiotherapy	<0.001		0.767	<0.001		0.199
No/unknown		Reference			Reference	
Yes		0.984 (0.882, 1.097)			0.932 (0.837, 1.038)	
Marital status	0.001		<0.001	0.001		<0.001
Unmarried		Reference			Reference	
Married		0.870 (0.808, 0.938)			0.871 (0.810, 0.936)	

CSS, cancer-specific survival; OS, overall survival; NI, not included in the multivariate survival analysis.

### Subgroup analysis of the effect of marital status in cancer-specific survival and overall survival

**FIGURE 3:**
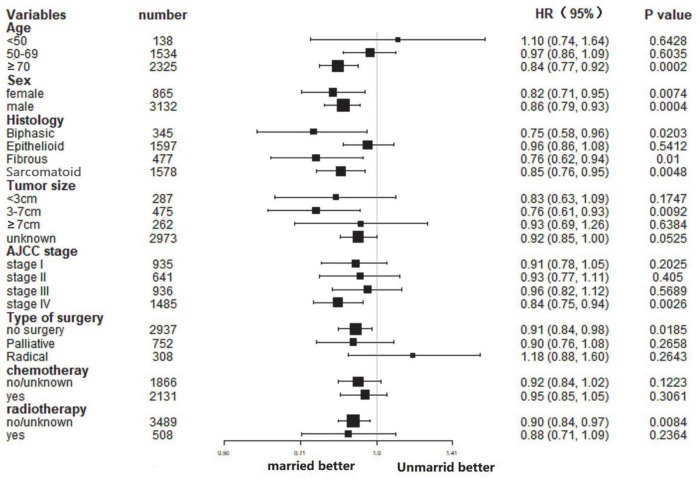
Forest plot of subgroup analysis for CSS.

**FIGURE 4:**
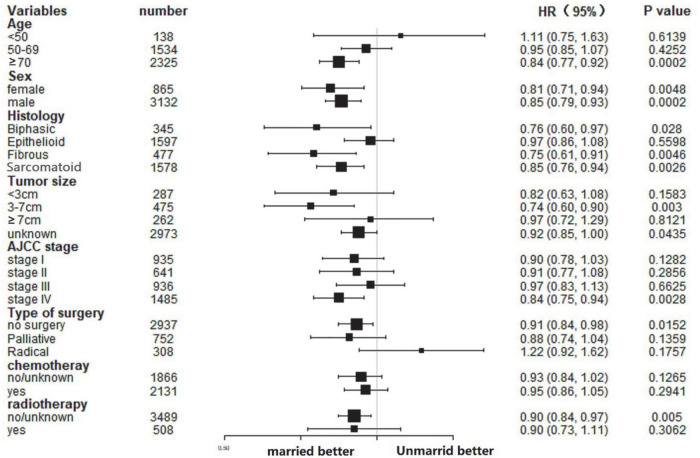
Forest plot of subgroup analysis for OS.

In this study, the influence of marital status on survival at different subgroups was analyzed. As demonstrated by the results of subgroup analysis, the married groups had better CSS and *OS* survival than the unmarried groups in nearly all study subgroups (Figures 3, 4). Specifically, the subgroups patients who aged ≥70, tumor size 3–7 cm, AJCC stage IV, and received no surgery or radiotherapy could significantly benefit from married status (all *p* < 0.05).

## Discussion

Marital status could be considered an independent prognostic indicator of MPM patients. This research probes into the impact of marital status on the survival of MPM patients for the first time. In this study of 3,997 patients with MPM, we observed that married groups had lower risk of death compared with unmarried ones. After the demographic and tumor features were controlled, the married patients had a 13% lower risk of death in comparison to unmarried ones.

Most cases of MPM are caused by prior exposure to asbestos (2, 5). Other causes of MPM include erionite (a mineral found in Turkish rocks), chest wall radiation, and simian virus 40. The latter, an oncogenic virus that blocks tumor suppressor genes, may be a cofactor in the development of MPM, although evidence for causality is weak (2, 5, 20–22).

There is an interaction between gender and marital status among a general population. According to the previous research, the impact of marital status on survival would vary with gender (23, 24). Hence, the stratification by gender was taken into account for subgroup analysis. Nevertheless, in comparison to the unmarried groups, the married groups presented better survival results at each gender subgroup. That is to say, in addition to gender, there was other etiology for the marital status’s impact on prognosis.

Some studies had hypothesized that unmarried people’s poor prognosis was related to the delayed diagnosis in tumor stage. For instance, a study on laryngeal and oral cancers revealed that married people were diagnosed with earlier stage cancer (25). However, we found that there were a slightly more stages I and II patients among unmarried groups. Moreover, many researchers hold opposite opinions through study (26, 27). As shown by the subgroup analysis results, the married status was an independent prognostic factor of MPM in stage IV disease, but not in stage I–III disease. In other words, marital status play an important role in advanced stage diseases, and is scilicet a protective factor in patients with advanced disease particularly.

Two potential mechanisms may explain the relationship between survival and marital status. First, after a diagnosis of cancer, the distress of married groups is less than that of unmarried ones, since a partner can help offer suitable support and share the stress (28). Moreover, distress and loneliness will trigger angiogenesis of tumor, cause down-regulation of cellular immune response (29), and lift invasiveness of tumor (30–32). Second, the married patients who gain support from their children or spouses could better comply with advice of doctors (33, 34). In this way, they are more likely to obtain active treatment. Likewise, our research discovered that married patients were more possibly to receive treatments. Hence, it is essential to provide social support services and psychological interventions that may help to lower the great survival differences between unmarried and married patients with cancer.

Nevertheless, owing to the SEER database’s limited nature, this study has a few limitations. Firstly, the marital status in this study was recorded at diagnosis. Hence, whether the changed marital status was unknown. Secondly, the detailed quality of marriage was not offered in SEER database, which would affect the survival results as well (35). Thirdly, there was a lack of more detailed data on education, insurance, and income, which may had a certain impact on the interpretation of the results. In order to verify these findings, prospective cohort studies are needed in the future. In spite of the above limitations, our research reveals that the marital status could significantly affect the survival of MPM. This study is meaningful, as it stresses the great impact of marriage, especially social support, on survival of cancer. At the same time, this study puts forward that offering social support interventions targeted at vulnerable people could help improve the possibility of being cured to a great extent. In the future research, this intervention may be confirmed a cost-effective way to improve outcomes among unmarried cancer patients.

## Conclusion

To sum up, the marital status is an independent prognostic indicator of MPM patients. Compared with unmarried patients, married ones are better in CSS and OS. Indeed, unmarried patients’ poor prognosis may be related to deficient treatment, socioeconomic and psychosocial factors. Further study is needed to confirm the finding of the existing study.

## Data availability statement

The original contributions presented in the study are included in the article/supplementary material, further inquiries can be directed to the corresponding author.

## Ethics statement

The Human Research Protection Office considered that the data analysis is focused on non-human subjects, and they were available. Hence, the approval from the institutional review board was not required. Written informed consent from the (patients/participants OR patients/participants legal guardian/next of kin) was not required to participate in this study in accordance with the national legislation and the institutional requirements.

## Author contributions

ZL conceived the study and revised the manuscript. SP searched the database and literature and wrote the manuscript. NY and YZ discussed and analyzed the data. All authors approved the final version.

## Abbreviations

MPM: malignant pleural mesothelioma
CSS: cancer-specific survival
OS: overall survival
HR: hazard ratio
CI: confidence interval
AJCC: American joint committee on cancer
TNM: tumor–node–metastasis
SEER: surveillance epidemiology and end results
ICD-O-3: international classification of disease for oncology third edition
K–M: Kaplan–Meier.
